# Integrated surveillance of extended-spectrum beta-lactamase (ESBL)-producing *Salmonella* and *Escherichia coli* from humans and animal species raised for human consumption in Canada from 2012 to 2017

**DOI:** 10.1017/S0950268822001509

**Published:** 2022-12-20

**Authors:** Courtney A. Primeau, Amrita Bharat, Nicol Janecko, Carolee A. Carson, Michael Mulvey, Richard Reid-Smith, Scott McEwen, Jennifer E. McWhirter, E. Jane Parmley

**Affiliations:** 1 Department of Population Medicine, Ontario Veterinary College, University of Guelph, Guelph, Ontario, Canada; 2 Centre for Food-borne, Environmental and Zoonotic Infectious Diseases, Public Health Agency of Canada, Guelph, Ontario, Canada; 3 National Microbiology Laboratory, Public Health Agency of Canada, Winnipeg, Manitoba, Canada; 4 Quadram Institute, Norwich, NR4 7UQ, UK

**Keywords:** Antimicrobial resistance, *Escherichia coli* (*E. coli*), *Salmonella*, surveillance, zoonotic foodborne diseases

## Abstract

Resistance to beta-lactam antimicrobials caused by extended-spectrum beta-lactamase (ESBL)-producing organisms is a global health concern. The objectives of this study were to (1) summarise the prevalence of potential ESBL-producing *Escherichia coli* (ESBL-EC) and *Salmonella* spp. (ESBL-SA) isolates from agrifood and human sources in Canada from 2012 to 2017, and (2) describe the distribution of ESBL genotypes among these isolates. All data were obtained from the Canadian Integrated Program for Antimicrobial Resistance Surveillance (CIPARS). CIPARS analysed samples for the presence of ESBLs through phenotypic classification and identified beta-lactamase genes (*bla*_TEM_, *bla*_SHV_, *bla*_CTX_, *bla*_OXA_, *bla*_CMY−2_) using polymerase chain reaction (PCR) and whole genome sequencing (WGS). The prevalence of PCR-confirmed ESBL-EC in agrifood samples ranged from 0.5% to 3% across the surveillance years, and was detected most frequently in samples from broiler chicken farms. The overall prevalence of PCR-confirmed ESBL-SA varied between 1% and 4% between 2012 and 2017, and was most frequently detected in clinical isolates from domestic cattle. The TEM-CMY2 gene combination was the most frequently detected genotype for both ESBL-EC and ESBL-SA. The data suggest that the prevalence of ESBL-EC and ESBL-SA in Canada was low (i.e. <5%), but ongoing surveillance is needed to detect emerging or changing trends.

## Introduction

Beta-lactam antimicrobials are one of the most widely used classes of antimicrobials in human and veterinary medicine, and resistance to these drugs is a global public health concern [1, 2]. Extended-spectrum cephalosporins and penicillins are important beta-lactams for treating and preventing infections caused by Gram-negative pathogens [1]. As a result of the extensive use of third- and fourth-generation cephalosporins, extended-spectrum beta-lactamase enzymes (ESBLs) capable of hydrolysing and conferring resistance to these antimicrobials emerged in people in both community and healthcare settings [1, 3, 4]. ESBL-producing Enterobacteriaceae have been increasing in prevalence in people worldwide, and are associated with adverse health outcomes and increased burden on health care systems [5, 6].

ESBLs pose a significant challenge to the effectiveness of infection control and antimicrobial stewardship efforts, as the genes encoding the ESBL enzymes are mostly located on or near mobile genetic elements, such as plasmids, insertion sequences, and transposons [7, 8]. The mobility of the genetic elements makes ESBL genes easily transmissible between individual bacteria, and even between bacteria of different species and genera [7, 8]. Furthermore, the genes coding for ESBL production are often carried on plasmids that carry additional antimicrobial resistance (AMR) factors, which contributes to the multi-class resistance of many ESBL-producing organisms [1]. For example, the CTX-M-15 enzyme is a common type of ESBL found globally in humans and agrifood sources, and has been located on specific plasmids that are also associated with other resistance determinants, particularly fluoroquinolone and aminoglycoside resistance genes [9, 10]. Infections caused by ESBL-producing organisms in both human and animal populations have also demonstrated resistance to ampicillin and trimethoprim-sulfamethoxazole, indicating that co-resistance may occur and must be considered when making treatment decisions and for infection control purposes [7].

Because of gene transfer within and between bacterial genera, it is difficult to characterise the transmission pathways of ESBL genes in bacterial populations and host species [7]. However, ESBL-producing organisms have been isolated from several food animal species and associated products, including domestic cattle, chickens, turkeys, and pigs, and some studies have suggested the existence of shared reservoirs of ESBL genes, plasmids and clones between animals and humans [11–13]. The evidence is less clear regarding potential transmission of ESBL isolates to humans from the food-chain. Some studies have demonstrated that ESBL-producing bacteria in food-producing animals and humans share a similar distribution of ESBL genes [14, 15], indicating that the food chain, specifically consumption of contaminated meat, could be an important route of transmission of ESBL genes and bacteria to humans. A study of ESBL-producing *E. coli* isolates from both human and poultry sources in the Netherlands found 19% of the human isolates contained the same ESBL genes as farm poultry isolates and 39% of ESBL-producing *E. coli* isolates found in retail chicken meat were associated with genotypes also found in the human samples [11]. This suggests that the transmission of ESBL *E. coli* may occur between live poultry, poultry products and humans, most likely through the food-chain [11]. It is also important to consider the importance of direct contact with animals potentially harbouring ESBL-producing organisms, as this could represent an additional transmission pathway between humans and animals.

To date, there have been few studies examining ESBL-producing Enterobacteriaceae across several human and animal sources in Canada. This study formed part of a doctoral thesis [16], and the objectives are to (1) summarise the prevalence of ESBL-producing *E. coli* (ESBL-EC) and *Salmonella* spp. (ESBL-SA) from human and agrifood sources in Canada from 2012 to 2017, and (2) describe the distribution of ESBL genotypes among these isolate.

## Methods

### Surveillance protocol for sample selection

All *E. coli* and *Salmonella* isolates were obtained from the Canadian Integrated Program for Antimicrobial Resistance Surveillance (CIPARS), a national surveillance programme that monitors human and animal antimicrobial use (AMU), and AMR in select bacteria from humans, animals and retail food [17]. This study included all isolates (*Salmonella* and *E. coli*) collected by CIPARS between 2012 and 2017 from the agrifood surveillance components, and between 2012 and 2016 for the human surveillance component, as the 2017 data were not available at the time of analysis. CIPARS uses active surveillance to obtain samples and associated risk factor information from farms, slaughter plants and from grocery stores. The CIPARS farm component uses Canadian sentinel farms to monitor AMU and AMR in broiler chickens, feedlot beef cattle, dairy cattle, grower-finisher pigs, and turkeys. The CIPARS farm broiler chicken component collects placement (flocks sampled at the time of chick placement on the farm) and pre-harvest (flocks sampled at least 1 week before shipment for slaughter) samples [17]. At abattoir, samples of caecal content are collected from Canadian federally inspected plants that slaughter chickens, pigs or cattle. The CIPARS retail component collects samples of raw chicken, beef, pork and turkey meat from grocery stores to test for AMR as a surrogate for potential human exposure to resistant bacteria through the consumption of meat. The retail meat samples originate from Canadian animal sources, or, in the case of beef and pork, may be imported from another country. The farm, abattoir and retail surveillance components monitor trends in AMR in *Salmonella*, *Campylobacter* and generic *E. coli*. Additionally, CIPARS tests *Salmonella* isolates from human and veterinary diagnostic submissions for resistance [17].

### Susceptibility and ESBL testing

**Fig. 1.**
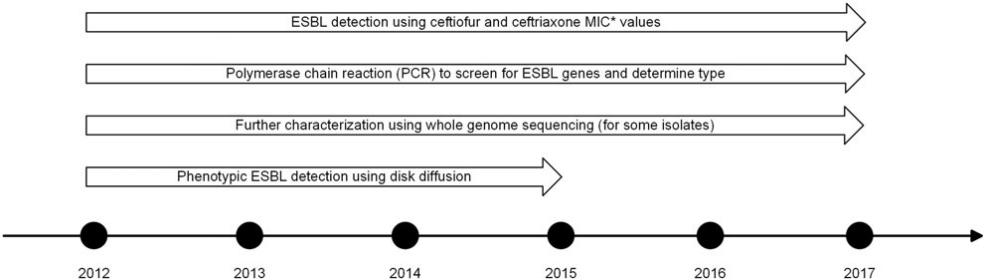
Schematic of the methods used for detection of potential extended-spectrum beta-lactamase-producing *E. coli* (ESBL-EC) and *Salmonella* spp. (ESBL-SA) by the Canadian Integrated Program for Antimicrobial Resistance Surveillance (CIPARS) during the period of 2012–2017, adapted from the primary author's doctoral thesis [16]. *MIC refers to the minimum inhibitory concentration.

The methods used for sample collection and antimicrobial susceptibility testing are described in detail in the CIPARS annual reports [17]. All susceptibility testing was completed by the National Microbiology Laboratory, Winnipeg, Manitoba (human clinical isolates), Guelph, Ontario (agrifood *Salmonella* isolates) or Saint-Hyacinthe, Québec (agrifood *E. coli* isolates). Briefly, minimum inhibitory concentration (MIC) values for *E. coli* and *Salmonella* were determined using a broth microdilution method, and susceptibility categories were interpreted according to Clinical and Laboratory Science Institute (CLSI) guidelines [18]. We defined potential ESBL-producers as any *E. coli* or *Salmonella* isolate with a ceftiofur MIC ≥4 μg/ml or a ceftriaxone MIC ≥0.5 μg/ml. Until 2016, we conducted phenotypic confirmation of ESBL-producers using CLSI confirmatory disk tests using disks containing cefotaxime, cefotaxime-clavulanic acid, ceftazidime and ceftazidime-clavulanic acid, in addition to polymerase chain reaction (PCR) testing [19]. In 2016 and 2017, potential ESBL-SA and ESBL-EC were confirmed by PCR and whole genome sequencing (WGS); no phenotypic confirmation was done. Genotype was determined using PCR on all isolates to detect *bla*_CMY−2_, *bla*_CTX−M_, *bla*_SHV_, *bla*_TEM_ and *bla*_OXA_. Isolates that lacked *bla*_CMY−2_, but contained a potential ESBL (*bla*_CTX−M_, *bla*_SHV_, *bla*_TEM_ or *bla*_OXA_) underwent WGS by the National Microbiology Laboratory (Winnipeg, Manitoba) to differentiate between the variants of the ESBL enzymes (Fig. 1). Sequencing was carried out on the MiSeq platform (Illumina, San Diego, CA, USA). A subset of *bla*_CMY−2_/*bla*_TEM_ isolates underwent sequencing on the NextSeq platform (Illumina). AMR prediction from whole genome sequences was carried out with the staramr tool [20]. When there were disagreements between the results by PCR and WGS, the results of WGS were used.

### Selective media testing

A subset of the CIPARS retail samples collected between 2012 and 2014 underwent additional testing for potential ESBL-producing Enterobacteriaceae using a selective media isolation approach. Frozen retail meat samples were thawed and enriched in 225 ml of buffered peptone water and incubated at 37°C for 18–24 h. Incubate broth was inoculated onto CHROMagar™ ESBL plates and incubated at 37°C for 18–24 h. Presumptive positive samples were subcultured onto MacConkey agar. Presumptive ESBL-producing colonies were further subcultured onto a tryptic soy agar plate and incubated. Disk diffusion phenotypic testing was then performed as described above. Presumptive isolates from meat samples were then submitted for phenotypic testing to the National Microbiology Laboratory (Guelph, Ontario) and further submitted for WGS testing at the National Microbiology Laboratory (Winnipeg, Manitoba).

### Data analysis

Surveillance data for each year were received from the laboratory in a Microsoft Excel 2016 spreadsheet. All isolate susceptibility and ESBL testing data were compiled into a summary spreadsheet, and all descriptive analyses were performed in Microsoft Excel 2016. An isolate was defined as ESBL-producing if it contained the *bla*_CTX−M_ or *bla*_OXA_ genes or if it was a known ESBL variant of *bla*_TEM_ or *bla*_SHV_; unfortunately, this was only possible to confirm for isolates that underwent WGS. Although we recognise that isolates containing the *bla*_CMY−2_ gene are not true ESBL producers, we classified isolates containing the *bla_C_*_MY−2_ gene with one of the true extended-spectrum beta-lactamase genes to be potential ESBL producers. The prevalence of ESBL-SA and ESBL-EC was calculated and defined as the total number of ESBL-EC or ESBL-SA isolates detected by PCR divided by the total number of samples submitted for testing for *E. coli* or *Salmonella*. The proportion of *E. coli* and *Salmonella* isolates that were potential ESBL-producers each year was also examined and was defined as the total number of ESBL-EC or ESBL-SA isolates detected genotypically (by PCR) divided by the total number of *E. coli* or *Salmonella* isolates recovered. Exact confidence intervals (Clopper Pearson confidence intervals, 95%) were calculated for all prevalence and proportion estimations. Summary tables were created for PCR-confirmed ESBL producers for both human and agrifood species. For both *Salmonella* and *E. coli*, the data were examined to identify any differences in the prevalence of agrifood ESBLs between years, regions and host species using univariable logistic regression in STATA [21]. Differences in the prevalence of human ESBLs between age classes, gender, region and year were also examined using univariable logistic regression.

## Results

### Prevalence of ESBL-EC

The total number of agrifood samples tested for *E. coli* by CIPARS between 2012 and 2017 was 25 353, and the total number of *E. coli* isolates recovered was 21 517. A total of 394 PCR-confirmed ESBL-EC were detected for an overall prevalence of 1.8% (95% CI 1.7–2.0) and an ESBL-EC proportion of 1.6% (95% CI 1.4–1.7; Table 1). The annual prevalence of potential ESBL-EC ranged between 0.3% and 2.2%. The highest annual prevalence of PCR-confirmed ESBL-EC was detected in 2016, with 93 PCR-confirmed ESBL-EC isolates identified and a prevalence of 2.2% (95% CI 1.8–2.8).

**Table 1.** The annual prevalence of PCR-confirmed extended-spectrum beta-lactamase producing *Escherichia coli* (ESBL-EC) for all agrifood samples obtained from the Canadian Integrated Program for Antimicrobial Resistance Surveillance (CIPARS) for the period of 2012–2017, adapted from the primary author's doctoral thesis [16]

Year	Total number of samples tested for *E. coli*	Total number of *E. coli* isolates recovered	Total number of *E. coli* isolates screened in phenotypically^a^	Total number of ESBL-EC detected by disk diffusion^b^	Total number of ESBL-EC detected by PCR	Prevalence of ESBL-EC (95% CI)^c^	Proportion of ESBL-EC (95% CI)^d^
2012	3586	3302	228	2	36	1.0% (0.7–1.4)	1.1 (0.8–1.5)
2013	3996	4187	229	18	77	1.9% (1.5–2.4)	1.8 (1.5–2.3)
2014	5349	4937	220	20	91	1.7% (1.4–2.1)	1.8 (1.5–2.3)
2015	4250	3030	119	14	14	0.3% (0.2–0.6)	0.5 (0.3–0.8)
2016	4168	3096	117	–	93	2.2% (1.8–2.8)	3.0 (2.5–3.7)
2017	4004	2965	68	–	83	2.1% (1.7–2.6)	2.8 (2.5–3.7)
Total	25 353	21 517	981	54	394	1.8% (1.7–2.0)	1.6 (1.4–1.7)

a Indicates the total number of *E. coli* isolates that have minimum inhibitory concentrations (MIC) of ≥4 mg/l for ceftiofur and ≥0.25 mg/l for ceftriaxone as determined by microbroth dilution for 2012–2015. In 2016 and 2017, only the MIC for ceftriaxone was used.

b Disk diffusion was not performed in 2016 or 2017.

c The prevalence of ESBL-EC for each year is defined as the total number of ESBL-EC isolates detected genotypically (by PCR) divided by the total number of samples submitted to CIPARS that year.

d The proportion of ESBL-EC for each year is defined as the total number of ESBL-EC detected genotypically (by PCR) divided by the total number of *E. coli* isolates recovered.

The largest proportion of PCR-confirmed ESBL-producing *E. coli* was detected in isolates recovered from chicken samples, with ESBL-EC detected across all chicken surveillance components (i.e. farm, abattoir and retail). The highest prevalence of PCR-confirmed ESBL-EC was in broiler chickens on farm (Table 2), with a prevalence of 5.7% (95% CI 4.5–7.1) at pre-harvest, in comparison to a prevalence of 4.7% (95% CI 3.9–5.6) detected among samples collected upon chick placement at farm. The next highest prevalence of PCR-confirmed ESBL-EC was found in retail chicken (3.4%, 95% CI 2.8–4.1), followed by chicken samples collected at abattoir (2.8%, 95% CI 2.0–4.0).

**Table 2.** The animal species distribution of PCR-confirmed extended-spectrum beta-lactamase producing *Escherichia coli* (ESBL-EC) for agrifood isolates obtained from the Canadian Integrated Program for Antimicrobial Resistance Surveillance (CIPARS) for the period of 2012–2017, adapted from the primary author's doctoral thesis [16]

CIPARS surveillance component	Species	Number of samples tested for *E. coli*	*E. coli* recovery rate (*n*)^a^	Number of ESBL-EC (% of all ESBL-EC)	Prevalence of ESBL-EC (95% CI)^b^
On-farm	Broiler chicken (placement)	1247	80.8% (1008)	71 (18.1)	5.7% (4.5–7.1)
	Broiler chicken (pre-harvest)	2408	99.2% (2389)	112 (28.6)	4.7% (3.9–5.6)
	Turkey	920	98.7% (908)	12 (3.1)	0.4% (0.2–0.7)
	Swine	3023	98.3% (2972)	32 (8.2)	1.1% (0.8–1.5)
Abattoir	Broiler chicken	1100	99.5% (1094)	31 (7.9)	2.8% (2.0–4.0)
	Swine	1059	99.0% (1048)	5 (1.3)	0.5% (0.2–1.1)
Retail	Chicken	2739	92.6% (2535)	93 (23.6)	3.4% (2.8–4.1)
	Turkey	2730	90.2% (2462)	24 (6.1)	0.9% (0.6–1.3)
	Beef	3954	49.9% (1974)	4 (1.0)	0.1% (0.04–0.3)
	Pork	4994	25.4% (1267)	10 (2.6)	0.2% (0.1–0.4)
Total		24 174	73.0% (17 657)	394 (100)	

a The recovery rate is defined as number of *E. coli* isolates recovered/number of samples submitted.

b The prevalence of ESBL-EC is defined as the number of PCR-confirmed ESBL-EC isolates detected/number of samples submitted.

### Effect of year, animal species and region on the prevalence of ESBL-EC

Univariable logistic regression models revealed no significant differences (*P* > 0.05) in the prevalence of potential ESBL-EC across years, animal species/food commodities, or sampling region in Canada.

### Molecular characterisation of ESBL-EC

Beta-lactamase genes identified in the ESBL-producing *E. coli* isolates from CIPARS agrifood surveillance are summarised in Table 3. Two hundred and forty-four (62%) of the ESBL-producing isolates carried multiple beta-lactamase genes, with TEM/CMY-2 (53%, 208/394) being the predominant genotype combination. The next most common genotypes were SHV-type gene in ESBL-EC (20.4%, 90/394) and *bla*_CTX−M_ containing ESBL-EC (13.0%, 51/394). Furthermore, a total of 163 potential ESBL-EC isolates underwent sequencing to further characterise the ESBL variants (Table 3). Of the 163 potential ESBL-EC isolates that underwent sequencing, 157 isolates were confirmed to be ESBL-EC. The most common genotype detected in the ESBL-EC isolates was SHV-2 (48.5%, 79/163), followed by CTX-M-1 (26.4%, 43/163) and SHV-1/TEM-1B (4.9%, 8/163), respectively. ESBLs CTX-M-14, −15 and −27, which are common in human-source *E. coli*, were also detected in this study in agrifood sources.

**Table 3.** The genotypic distribution of PCR-confirmed extended-spectrum beta-lactamase producing *Escherichia coli* (ESBL-EC) and extended-spectrum beta-lactamase producing *Salmonella* spp. (ESBL-SA) for agrifood isolates obtained from the Canadian Integrated Program for Antimicrobial Resistance Surveillance (CIPARS) for the period of 2012–2017, adapted from the primary author's doctoral thesis [16]

ESBL-EC				ESBL-SA			
PCR results only (*n* = 394)		WGS results (*n* = 163)		PCR results only (*n* = 263)		WGS results (*n* = 38)	
Genotype	*n* (% of all ESBL-EC)	Genotype	*n* (% of all sequenced ESBL-EC)	Genotype	*n* (% of all ESBL-SA)	Genotype	*n* (% of all sequenced ESBL-SA)
*bla*_TEM/CMY−2_	208 (53.1)	bla_SHV−2_	79 (48.5)	*bla*_TEM/CMY−2_	210 (74.5)	*bla*_SHV−2_	14 (5.3)
*bla*_SHV_	80 (20.4)	*bla*_CTX−M−1_	43 (26.4)	PCR-^a^	19 (6.7)	*bla*_CTX−M−1_	13 (4.9)
*bla*_CTX−M_	51 (13.0)	*bla*_SHV−2/TEM−1B_	8 (4.9)	*bla*_SHV_	16 (5.7)	*bla*_SHV−12/TEM−1B/CMY−2_	4 (1.5)
*bla*_TEM_	18 (4.6)	*bla*_SHV−2/CMY−2_	7 (4.3)	*bla*_CTX−M_	14 (5.0)	*bla*_CTX−M−1/CMY−2_	2 (0.8)
*bla*_SHV/TEM_	12 (3.1)	*bla*_CTX−M−55_	4 (2.5)	*bla*_OXA/CMY−2_	6 (2.1)	*bla*_SHV−12/TEM−1B_	2 (0.8)
*bla*_TEM/CTX−M_	8 (2.0)	*bla*_TEM−52B_	3 (1.8)	*bla*_SHV/TEM/CMY−2_	4 (1.4)	*bla*_CTX−M−1/TEM−1A_	1 (0.4)
*bla*_SHV/CMY−2_	7 (1.8)	*bla*_CTX−M−1/CMY−2_	3 (1.8)	*bla*_TEM/CTX−M_	4 (1.4)	*bla*_CTX−M−1/TEM−1B_	1 (0.4)
*bla*_CTX−M/CMY−2_	3 (0.77)	*bla*_CTX−M−1/TEM−1A_	2 (1.2)	*bla*_TEM_	3 (1.1)	*bla*_CTX−M−55/TEM−1B_	1 (0.4)
*bla*_OXA/CMY−2_	2 (0.51)	*bla*_CTX−M−124_	1 (0.61)	*bla*_CTX−M/CMY−2_	2 (0.7)		
*bla*_SHV/TEM/CMY−2_	2 (0.51)	*bla*_CTX−M−15_	1 (0.61)	*bla*_SHV/TEM_	2 (0.7)		
*bla*_OXA/CTX−M_	1 (0.26)	*bla*_CTX−M−27_	1 (0.61)	*bla*_SHV/TEM/CTX−M/CMY−2_	1 (0.4)		
		*bla*_CTX−M−65_	1 (0.61)				
		*bla*_CTX−M−15/OXA−1_	1 (0.61)				
		*bla*_SHV−2/TEM−1B/CMY−2_	1 (0.61)				
		*bla*_TEM−88_	1 (0.61)				
		*bla*_SHV−2/TEM−1C/CMY−2_	1 (0.61)				

a PCR- indicates isolates that tested positive phenotypically using the disk diffusion test, but did not have ESBL genes detected during PCR.

### Selective media results

Using selective media, we identified an additional nine ESBL-EC isolates from the retail surveillance component of CIPARS (Table 4); none of these nine isolates had been detected through the non-selective media isolation methodology. Most of the additional isolates (67%, 6/9) were collected from retail chicken samples, with the remaining ESBL-EC detected in retail pork (1/9), turkey (1/9) and beef (1/9). The most common genotype observed in the ESBL-EC isolated using selective media was TEM-52B (33.3%, 3/9), followed by CTX-M-1 (22.2%, 2/9).

**Table 4.** The distribution by sampled animal species/food commodity of the nine extended-spectrum beta-lactamase producing *Escherichia coli* (ESBL-EC) isolates collected from the retail surveillance component of the Canadian Integrated Program for Antimicrobial Resistance Surveillance (CIPARS) between 2012 and 2014, and isolated using a selective media methodology, adapted from the primary author's doctoral thesis [16]

Species (*n*)	Genotype (*n*)
Retail chicken (6)	*bla*_TEM−52B_ (3)
	*bla*_CTX−M−1_ (1)
	*bla*_CTX−M−55_ (1)
	*bla*_SHV−2/CMY−2_ (1)
Retail pork (1)	*bla*_CTX−M−15/TEM−1B_
Retail beef (1)	*bla*_CTX−M−15/OXA−1_
Retail turkey (1)	*bla*_CTX−M−1_
Totals (*n* = 9)	*bla*_TEM−52B_ (3)
	*bla*_CTX−M−1_ (2)
	*bla*_CTX−M−15/TEM−1B_ (1)
	*bla*_CTX−M−15/OXA−1_ (1)
	*bla*_SHV−2/CMY−2_ (1)
	*bla*_CTX−M−55_ (1)

### Prevalence of ESBL-SA

CIPARS tested a total of 28 552 agrifood samples for *Salmonella* between 2012 and 2017, and a total of 13 461 *Salmonella* isolates were recovered. Among those isolates, 263 PCR-confirmed ESBL-SA were detected, yielding an overall prevalence of 0.9% (95% CI 0.8–1.0). Overall, 2% of the *Salmonella* isolates collected between 2012 and 2017 were potential ESBL-producers. The annual prevalence of potential ESBL-SA ranged between a low of 0.5% (95% CI 0.3–0.8) in 2012 and a high of 1.6% (95% CI 1.3–1.9) in 2014 (Table 5).

**Table 5.** The annual prevalence of potential extended-spectrum beta-lactamase producing *Salmonella* spp. (ESBL-SA) for agrifood isolates obtained from the Canadian Integrated Program for Antimicrobial Resistance Surveillance (CIPARS) for the period of 2012–2017, adapted from the primary author's doctoral thesis [16]

Year	Total number of samples tested for *SA*	Total number of *SA* isolates recovered	Total number of *SA* Isolates screened in phenotypically^a^	Total number of ESBL-SA detected by disk diffusion^b^	Total number of ESBL-SA detected by PCR	Prevalence of ESBL-SA (95% CI)	Proportion of ESBL-SA (95% CI)^d^
2012	4017	2337	442	110	24	0.6% (0.4–0.9)	1.0% (0.7–1.5)
2013	4562	2210	503	123	44	1.0% (0.7–1.3)	2.0% (1.5–2.7)
2014	5782	2212	323	163	91	1.6% (1.3–1.9)	4.1% (3.3–5.0)
2015	4810	2450	303	103	30	0.6% (0.4–0.9)	1.2% (0.9–1.7)
2016	4786	2221	191	–	51	1.1% (0.8–1.4)	2.3% (1.8–3.0)
2017	4595	2031	167	–	23	0.5% (0.3–0.8)	1.1% (0.8–1.7)
Total	28 552	13 461	1929	499	263	0.9% (0.8–1.0)	2.0% (1.7–2.2)

a Indicates the total number of *Salmonella* isolates that have minimum inhibitory concentrations (MIC) of ≥4 mg/l for ceftiofur and ≥0.25 mg/l for ceftriaxone as determined by microbroth dilution for 2012–2015. In 2016 and 2017, only the MIC for ceftriaxone was used.

b Disk diffusion was not performed in 2016 or 2017.

^c^The prevalence of ESBL-SA for each year was defined as the total number of ESBL-SA detected genotypically (by PCR) divided by the total number of samples submitted to CIPARS for that year.

d The proportion of ESBL-SA for each year was defined as the total number of ESBL-SA detected genotypically (by PCR) divided by the total number of *Salmonella* isolates recovered.

The highest percentage of PCR-confirmed ESBL-SA were detected among clinical isolates collected by passive surveillance (91%), and diagnostic cases from domestic cattle (*n* = 187) accounted for 71% of the total ESBL-SA detected (Table 6). Very few ESBL-SA (*n* = 24, 9.1% of all *Salmonella* isolates) were detected among samples collected from CIPARS active surveillance components; among these components, the highest prevalence of potential ESBL-SA appeared in isolates collected from turkey farms (0.7%; 95% CI 0.3–1.4) and swine farms (0.2%; 95% CI 0.07–0.4).

**Table 6.** The animal species distribution of potential extended-spectrum beta-lactamase producing *Salmonella* spp. (ESBL-SA) for agrifood isolates obtained from the Canadian Integrated Program for Antimicrobial Resistance Surveillance (CIPARS) for the period of 2012–2017, adapted from the primary author's doctoral thesis [16]

CIPARS surveillance component	Species	Number of samples tested for *Salmonella*	*Salmonella* recovery rate (*n*)^a^	Number of ESBL-SA (% of all ESBL-SA)	Prevalence of ESBL-SA (95% CI)
On-farm	Broiler chicken	3562	39.5% (1441)	1 (0.4)	0.03% (0.004–0.2)
	Turkey	920	45.7% (420)	6 (2.3)	0.7% (0.3–1.4)
	Swine	3042	22.6% (686)	5 (1.9)	0.2% (0.07–0.4)
Abattoir	Broiler chicken	4373	16.2% (709)	2 (0.8)	0.05% (0.01–0.2)
	Swine	2112	50.2% (1060)	1 (0.4)	0.05% (0.008–0.3)
Retail	Chicken	5002	32.0% (1600)	5 (1.9)	0.1% (0.04–0.2)
	Turkey	4437	19.8% (878)	4 (1.5)	0.09% (0.04–0.2)
	Pork	4995	1.6% (81)	0 (0)	–
Diagnostic cases/passive surveillance	Domestic cattle			187 (71.1)	
	Turkey			20 (7.6)	
	Swine			20 (7.6)	
	Chicken			3 (1.1)	
	Other (dog, horse, mink, cat, unspecified bird)			9 (3.4)	
Total		28 443	24.2% (6875)	263	

a The bacterial recovery rate is defined as number of *Salmonella* isolates recovered/number of samples submitted.

CIPARS tested 13 894 human clinical *Salmonella* isolates for ESBL-production between 2012 and 2016 (Table 7). Over this study period, the numbers of ESBL-SA remained very low, with 0.4% (*n* = 82) of all *Salmonella* isolates identified as potential ESBL-producers.

**Table 7.** The annual proportion of human clinical *Salmonella* spp. cases that are potential ESBL-producers obtained from the Canadian Integrated Program for Antimicrobial Resistance Surveillance (CIPARS) for the period of 2012–2016, adapted from the primary author's doctoral thesis [16]

Year	Total number of *Salmonella* isolates screened	Total number of ESBL-SA detected by disk diffusion	Total number of ESBL-SA detected by PCR	Proportion of ESBL-SA (%)^†^
2012	3645	18	14	0.38
2013	2940	19	10	0.34
2014	2544	7	6	0.24
2015	2360	11	10	0.42
2016	2405	2	2	0.08
Total	13 894	57	42	0.30

^a^The proportion of ESBL-SA for each year was defined as the total number of ESBL-SA detected genotypically (by PCR) divided by the total number of *Salmonella* isolates screened.

### Effect of year, animal species and region on the prevalence of ESBL-SA

Univariable logistic regression models revealed no significant differences (*P* > 0.05) in the prevalence of potential ESBL-SA across years, animal species/food commodities or sampling region in Canada.

### Effect of year, gender, age class and location on human ESBL-SA detection

Univariable logistic regression models were used to examine differences in the prevalence of potential ESBL-SA in humans across years, gender, region in Canada and age class. There were no differences (*P* > 0.05) in the prevalence of potential ESBL-SA detected between years, gender, region or age class.

### Molecular characterisation of ESBL-SA

Beta-lactamase genes identified in the ESBL-producing *Salmonella* spp. isolates from CIPARS agrifood surveillance are summarised in Table 3. Of the 263 total potential ESBL-SA isolates detected over 2012–2017, 81.6% contained multiple beta-lactamase genes. The most frequently identified genotype combination was TEM/CMY-2 (74.5%, 210/263), followed by SHV (5.7%, 16/263) and CTX-M (5.0%, 14/263). The genotypes of 38 ESBL-SA isolates were further characterised using WGS (Table 3). Of these 38 isolates, the principal genotype was SHV-2 (39.5%, 14/38), followed by CTX-M-1 (34.2%, 13/38). Additionally, four phenotypically identified ESBL-SA clinical isolates from domestic cattle carried two beta-lactamase genes (SHV-12 and TEM-1B), in addition to CMY-2.

A total of 15 different genotypes were identified in the ESBL-SA isolates from human clinical cases (Table 8), with the most common being CTX-M-65, followed by SHV-2/TEM-1.

**Table 8.** The genotypic distribution of potential extended-spectrum beta-lactamase producing *Salmonella* spp. (ESBL-SA) from human isolates obtained from the Canadian Integrated Program for Antimicrobial Resistance Surveillance (CIPARS) for the period of 2012–2016

Genotype	*n* (% of all ESBL-SA)
*bla*_CTX−M−65_	9 (21.4)
*bla*_SHV−2/TEM−1_	8 (19.0)
*bla*_SHV−2_	4 (9.5)
*bla*_CTX−M−1_	3 (7.1)
*bla*_CTX−M−15_	3 (7.1)
*bla*_CTX−M−9_	3 (7.1)
*bla*_CTX−M−14_	2 (4.8)
*bla*_CTX−M−55_	2 (4.8)
*bla*_CTX−M−55/TEM−1_	2 (4.8)
*bla*_CTX−M−14/TEM−1_	1 (2.4)
*bla*_CTX−M−15/OXA−1/TEM−1_	1 (2.4)
*bla*_SHV−5_	1 (2.4)
*bla*_CTX−M−14/CMY−2_	1 (2.4)
*bla*_CTX−M−3_	1 (2.4)
*bla*_CTX−M−65/CMY−2_	1 (2.4)

All genotypes were confirmed using whole genome sequencing, adapted from the primary author's doctoral thesis [16].

## Discussion

To the best of our knowledge, these are the first published data on the frequency and distribution of PCR-confirmed ESBL-EC and ESBL-SA data from the various CIPARS surveillance components and this is the first study examining the prevalence of ESBL-SA across animals, food and humans in Canada. Overall, the results of this study highlight a relatively low prevalence (<5%) of ESBL-EC in animals and meat and ESBL-SA among animal, meat and human isolates in Canada, but several different ESBL genotypes were identified across species and surveillance components.

The CIPARS surveillance data demonstrated that the prevalence of ESBL genes in broiler chickens and chicken meat in Canada is generally lower than rates reported by other countries, although prevalence rates of ESBL producers from broiler chickens in the literature vary widely by region. A German study found a prevalence of ESBL-producing Enterobacteriaceae of 1.7% in cloacal and environment samples in seven broiler flocks upon chick arrival at the farm [22]. In contrast, a Dutch study of 50 broiler farms found a pooled prevalence of 80% or higher on 85% of farms sampled using selective media [23]. Additionally, a Japanese study investigating ESBL-producing *E. coli* across food animal species highlighted a prevalence of 60% among individual broiler rectal isolates [24]. Differences in sampling methods, year, bacterial species being investigated, and testing methodologies make comparisons across studies challenging; however, there are clear differences in the prevalence between geographic regions, which could be a result of differing AMU or other management practices on farms. Because genes encoding ESBLs are often found on plasmids carrying other resistance genes, overall use of antimicrobials may play an important role in the ESBL prevalence differences observed among different countries. In mid-2014, the Canadian poultry industry implemented a national ban on the use of antimicrobials of very high importance to human medicine (Category I) [25] for disease prevention purposes [26, 27]. By the end of 2018, preventive use of antimicrobials of high importance to human medicine was also banned [26, 27]. Since 2015, no broiler flocks participating in CIPARS farm surveillance have reported any use of the third-generation cephalosporin ceftiofur [17, 28]. Since the ban on preventive use of Category I and II antimicrobials in broiler chickens and turkeys, fewer *E. coli* and *Salmonella* isolates recovered from broiler chickens on farm, at abattoir, and at retail have demonstrated resistance to ceftriaxone (another third-generation cephalosporin) [17]. Adoption of these antimicrobial reduction initiatives may have contributed to the low prevalence of ESBL-EC and ESBL-SA in chicken and turkey over the years included in this study. The prevalence of ESBL genes in retail beef and pork were also very low over the years included in this study, with no ESBL-SA detected in retail pork or beef. The use of Category I antimicrobials (i.e. ceftiofur) was reported in grower-finisher pig herds participating in CIPARS between 2012 and 2017 [17, 29–33]. Between 2012 and 2016, 18–20% of grower-finisher herds participating in CIPARS reported the use of ceftiofur [17, 29–32]. In 2017, 9% of grower-finisher herds participating in CIPARS reported the use of ceftiofur [33]. Unfortunately, there are no AMU data available for beef over this reporting period.

It may also be important to consider other farm-related factors that may account for differences in prevalence of ESBL-producing organisms. Several farm management factors, such as exposure to contaminated water or feed, water acidification and type of production system (i.e. organic *vs.* conventional) may facilitate the transmission of ESBL-producing organisms [34]. Therefore, differences between farm management in Canada and other geographical regions may at least partly account for differences in the prevalence of ESBL-EC detected from surveillance data, and these differences make it difficult to compare Canadian ESBL-EC prevalence to other countries.

In total, 23% (93/394) of the potential ESBL-EC isolates detected by CIPARS over the study period were found in retail chicken. The overall prevalence of potential ESBL-EC in retail chicken was 3.4% (95% CI 2.8–4.1) using traditional culture methods, which is relatively low compared to data published in other regions. For example, 37% of retail chicken samples in a study in Germany were ESBL-EC positive [35], and a French study found a prevalence of 91.7% in retail chicken [36].

Despite the low prevalence of potential ESBL-EC demonstrated in farm broiler chicken and retail chicken in Canada, ongoing surveillance and research is needed, as many Canadians are exposed to bacteria from chicken through the food chain, and chicken may represent an important reservoir for ESBL-producing organisms and ESBL genes [14, 37, 38]. ESBL genes are often located on plasmids that may be transmitted within and between populations, and the presence of these genes has been documented in chicken [11]. In addition to the potential exposure through broiler chicken consumption, direct contact with chickens or their production environment could represent an alternative transmission route for ESBL-producing bacteria. A study in the Netherlands found that individuals on broiler farmers had a higher prevalence of ESBL- and AmpC beta-lactamase producing *E. coli* carriage compared to the general population [37]. The authors also found a positive association between individuals having a high degree of contact with live broiler chickens and ESBL- and AmpC beta-lactamase producing *E. coli* carriage. Considering the mobility of ESBL genes and the potential for humans to be exposed through the food chain or direct contact, this highlights an important area for ongoing surveillance.

In our study, the use of a selective medium identified six additional isolates containing ESBL genes in retail chicken. This slightly increased the prevalence of potential ESBL-EC in over this study period, suggesting that the choice of medium could have important implications for the recovery of ESBL-EC and the ongoing surveillance of these organisms. Future surveillance efforts should carefully consider the choice of methods used for phenotypic and/or molecular detection of ESBL-producing organisms among samples. Although appropriateness of the methods will depend on the objectives of the surveillance system, selective media can improve detection of microorganisms that are present in low numbers [39]. Previous studies have demonstrated that using selective media can enhance the detection of ESBL-EC in rectal and faecal samples collected from hospitalised patients [40–42]. A study of rectal swabs from over 500 patients in a Dutch hospital concluded that 25% of ESBL-EC rectal carriers were identified only by a selective medium, and were not detected by non-selective testing [43]. Similar results were observed in a study of stool samples collected from hospitalised patients in Germany, and highlighted the added value of a pre-enrichment step, as 31% of ESBL-EC carriers were identified only by pre-enrichment [42]. Furthermore, use of selective media may be a valuable addition to standard monitoring for AMR organisms in food-producing species [44]. A 2012 study demonstrated that the use of selective media identified ESBL-EC in pigs on farm, retail chicken, pork and beef that were not identified when screening for ESBL-EC without selective media [44].

Despite the evidence that selective media may enhance the sensitivity of diagnostic screening, the use of ESBL-detecting selective media is not common practice in clinical or research settings, and the benefits of using this approach are still controversial and debated [42, 43]. This approach is more time-consuming, resulting in higher costs and required resources [42, 43]. Surveillance programmes usually carry out susceptibility testing on a variety of drug classes, not just beta-lactam drugs. Additionally, a recent study raised concerns that the use of selective media may be unreliable, and suggested that the selective enrichment method selects for TEM-type ESBLs, resulting in a potentially inflated detection level of TEM-type ESBL producers in samples [45]. Despite these potential limitations, the added value of selective media in detecting ESBL-producing organisms should continue to be investigated. The selective media results of this study suggest that ESBL-producing bacteria are present in our surveillance samples, but at very low levels below the detection limit of standard methods. These levels may not be sufficient to cause disease in humans or animals, but could still colonise a host and result in disease at a later data if exposed to AMU or other selection pressures. Therefore, it is still important to monitor these bacteria and identify potential trends. The prevalence of ESBLs is likely higher than our standard surveillance methods indicate, and the use of selective media could be a beneficial surveillance supplement to ensure that ESBL-producing organisms are being identified early to best trigger action to limit transmission between and within populations. Although standard culture methods may underestimate the prevalence of ESBL producers, these methods provide a robust framework for assessing trends in resistance, and if ESBL detection increases, further testing and new detection methods could be added.

The prevalence of potential ESBL-SA was highest among clinical isolates collected from domestic cattle. Clinical isolates are recovered from animals that would not be immediately entering the food chain and would therefore not present the same risk to public health compared to isolates from healthy animals on farm or at slaughter. However, these animals could potentially be a source of environmental contamination, and transfer ESBL-SA to other animals in the herd that may be entering the food chain or come into direct contact with humans, so it is important to continue to monitor clinical isolates. In contrast, the prevalence of potential ESBL-SA among food animal isolates on farm, at abattoir and at retail was very low. There are few studies investigating the prevalence of ESBL-SA in food-producing species compared to ESBL-EC, hence it is challenging to compare our results to other findings. A 2014 study of *Salmonella enterica* in healthy chickens and swine in Belgium highlighted that 96.6% of chicken-derived *Salmonella* isolates carried an ESBL gene, and 71.4% of swine-derived *Salmonella* isolates carried an ESBL gene [46]. Our data indicate a much lower prevalence of ESBL-SA overall, and a lower prevalence of ESBL-SA in broilers compared to pig isolates collected on farms. However, the total number of ESBL-SA isolates was still very low in both species over the study period, with one isolate detected from broiler chickens on farm, and five ESBL-SA isolates detected from pigs on-farm. A previous study of 32 randomly selected broiler farms concluded that the *β*-lactam antimicrobial amoxicillin is the most frequently used antimicrobial in Belgian broiler production, with 43% of sampled farms reporting use [47]. In contrast, the most commonly used antimicrobial on broiler chicken farms participating in CIPARS in 2016 was bacitracin, and no farms reported the use of any of Health Canada's Category I antimicrobials that year (third generation cephalosporins or fluoroquinolones) [17]. Furthermore, a 2017 study of *Salmonella* contamination in retail chicken in South Korea found that 63.6% of *Salmonella* isolates from conventionally raised broiler chicken were ESBL-producers [48], which is substantially higher than the prevalence observed in isolates obtained from Canadian retail chicken samples. However, the South Korean study was based on a small sample size and may not be reflective of the prevalence in retail chicken across South Korea. Overall, it appears that the prevalence of ESBL-SA in Canada is lower compared to ESBL-EC across the agrifood surveillance components, with very few isolates collected over the study period.

The annual proportion of potential ESBL-SA among human clinical isolates collected by CIPARS ranged between 0.11% and 0.72%, with an overall prevalence of 0.39% between 2012 and 2016. These data are similar to reports from the National Antimicrobial Resistance Monitoring System for Enteric Bacteria (NARMS) in the United States, which reported ESBL-SA prevalence of 0.34% and 0.28% among human clinical isolates in 2014 and 2015, respectively [49, 50]. The distribution of ESBL genes was also similar between CIPARS and NARMS. In the United States in 2015, ceftriaxone resistance in human isolates was most commonly conferred by CTX-M and SHV.

Importantly, the most common potential ESBL-SA genotype in this study was TEM-CMY2 among both the human clinical and agrifood isolates. This highlights the One Health nature of the issue of AMR, and the potential for humans and animals to share similar genes encoding resistance to the critically important drugs, such as *β*-lactam antimicrobials. This also undermines the importance of integrated surveillance of ESBLs and other important resistance determinants in humans and along the food chain. However, this does not necessarily represent direct evidence for the zoonotic transfer of ESBL genes through food contamination. The majority of the TEM-CMY2 isolates were from clinical *Salmonella* cases in domestic cattle rather than from animals close to slaughter or from retail meat; any spread of ESBL genes from clinically sick animals to humans is more likely through direct contact or via the environment than through food.

A potential limitation of our study is the relatively small number of isolates that underwent further molecular characterisation via WGS. Although PCR results can help inform which ESBL genes are circulating in each of the host species examined, the specific genotype (e.g. TEM-1 *vs.* TEM-2) cannot be determined without further analysis such as sequencing, making it challenging to determine whether these organisms are true ESBL-producers. Additionally, PCR only identifies the ESBL genes for which primers are available, so novel or rare ESBL gene variants may not be detected using only PCR. WGS can produce more precise information on the specific genes and plasmids compared to PCR, which can help gain greater insight into different transmission routes and the complex epidemiology of ESBL-producing Enterobacteriaceae, including molecular relatedness of isolates collected from different species [43]. Sequencing has important implications for surveillance, diagnostics and infection control [51]. Obtaining more precise results more quickly will be beneficial when monitoring trends in ESBL-producing bacteria and can also help identify uncommon or novel ESBL genes that are circulating. The molecular relatedness of isolates can help inform epidemiological investigations to determine the source of ESBL outbreaks and identify the drivers contributing to the emergence of ESBLs, which is important knowledge for the implementation of interventions. An improved understanding of the transmission routes of ESBL genes will be useful for developing potential interventions and policy changes to reduce the presence of ESBL-producers. The ability to monitor the prevalence of ESBL-encoding genes across bacteria isolated from humans and food-producing animals can be used to rapidly identify emerging issues and help implement timely control strategies. The diversity of ESBL genes identified in this study highlights the need for ongoing surveillance of the genes circulating in animals, food and humans, as this information will be critical for identifying areas to intervene.

In conclusion, this study highlighted the relatively low prevalence of potential ESBL-EC and ESBL-SA across animals, food and humans in Canada. Between 2012 and 2017, the total number of ESBL-EC and ESBL-SA isolates obtained from CIPARS surveillance generally decreased, though this trend was not statistically significant. This may be a result of the 2014 poultry industry initiative that banned the preventative use of ceftiofur in chicken, as this has resulted in a decrease in AMU and AMR in poultry and people in Canada [28]. Despite the low prevalence detected, ongoing surveillance across the farm-to-fork continuum and humans is needed to detect emerging trends, as ESBL-producing organisms can pose significant treatment challenges in both human and veterinary medicine. Additionally, future research should continue to identify potential risk factors for ESBL-producing organisms in animal species and humans in Canada to identify priority areas for interventions.

## Data Availability

Data are available from the Public Health Agency of Canada. Note: An earlier version of this manuscript is included in the corresponding author's doctoral thesis entitled ‘Exploring the contributions of genotypic, phenotypic, social and qualitative data sources to our understanding of antimicrobial resistance in Canada’ [16].
